# Unlocking the Potential of Mesenchymal Stem Cells in Gynecology: Where Are We Now?

**DOI:** 10.3390/jpm13081253

**Published:** 2023-08-13

**Authors:** Ivana Erceg Ivkošić, Rajko Fureš, Vesna Ćosić, Nika Mikelin, Luka Bulić, Domagoj Dobranić, Petar Brlek, Dragan Primorac

**Affiliations:** 1 St. Catherine Specialty Hospital, 10000 Zagreb, Croatialuka.bulic0302@gmail.com (L.B.);; 2 Faculty of Dental Medicine and Health, Josip Juraj Strossmayer University of Osijek, 31000 Osijek, Croatia; 3 Department of Gynecology and Obstetrics, Zabok General Hospital and Croatian Veterans Hospital, 49210 Zabok, Croatia; 4 Poliklinika Ćosić, d.o.o., 35000 Slavonski Brod, Croatia; 5 Health Center of the Zagreb County, 10000 Zagreb, Croatia; 6 School of Medicine, JJ Strossmayer University of Osijek, 31000 Osijek, Croatia; 7 The Henry C. Lee College of Criminal Justice and Forensic Sciences, University of New Haven, West Haven, CT 06516, USA; 8 Medical School, University of Split, 21000 Split, Croatia; 9 Eberly College of Science, The Pennsylvania State University, University Park, State College, PA 16802, USA; 10 Medical School, University of Rijeka, 51000 Rijeka, Croatia; 11 Medical School REGIOMED, 96 450 Coburg, Germany; 12 Medical School, University of Mostar, 88000 Mostar, Bosnia and Herzegovina

**Keywords:** mesenchymal stem cell, gynecology, regenerative medicine, infertility, lichen sclerosus, polycystic ovary syndrome, premature ovarian insufficiency, genitourinary syndrome of menopause, rectovaginal fistula

## Abstract

Stem cells, with their remarkable capacity for differentiation into diverse cell types, are vital for the development as well as maintenance of health and homeostasis. Two unique abilities set them apart from other cells: self-renewal and the capacity for differentiation. They play important roles in embryogenesis, development, regeneration, and various other processes. Over the last decade, there has been increased interest in their potential use in the treatment of numerous diseases and disorders across multiple fields of medicine in acute, chronic, innate, and acquired diseases. Stem cells are key to maintaining the body’s homeostasis and regulating growth and tissue functions. There are several types of stem cells—embryonic, adult, and human-induced pluripotent cells. Currently, mesenchymal stem cells are of great interest due to their regenerative, immunomodulatory, analgesic, and antimicrobial (anti-inflammatory) effects. Recent studies have shown the potent regenerative effect of stem cell therapy in gynecologic diseases such as infertility, Asherman syndrome, lichen sclerosus, polycystic ovary syndrome, premature ovarian insufficiency, genitourinary syndrome of menopause, and rectovaginal fistulas. Moreover, the successful isolation of oogonial stem cells could lead to a revolution in the field of gynecology and the potential treatment of the conditions discussed. This review aims to provide a better understanding of the latest therapeutic options involving stem cells and raise awareness of this promising yet not widely known topic in gynecology and medicine in general.

## 1. Introduction

**Figure 1:**
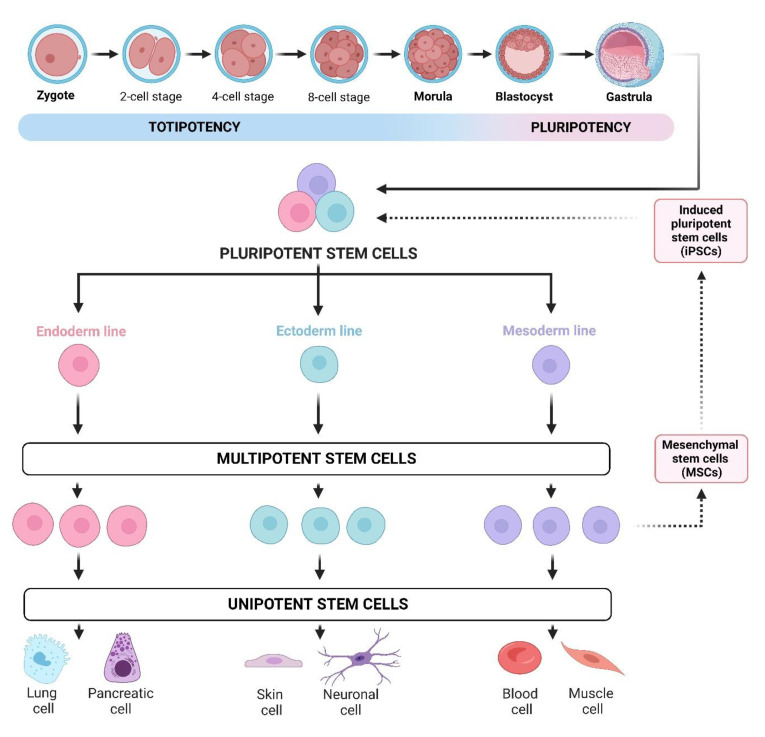
Levels of stem cell differentiation potential: Totipotent stem cells possess the highest potential, as they can generate all cell types in the body, including those found in embryos and supportive tissues such as the placenta. Pluripotent stem cells are slightly more specialized and can differentiate into cells from the three germ layers—ectoderm, endoderm, and mesoderm—which give rise to various tissues and organs. These cells are present in embryos during the blastocyst stage and certain adult tissues. Multipotent stem cells have a more limited potential compared to totipotent and pluripotent stem cells. They can differentiate into multiple cell types within a specific lineage or tissue type. Mesenchymal stem cells (MSCs), for example, are multipotent stromal cells capable of differentiating into a range of cell types, including osteoblasts, chondrocytes, myocytes, and adipocytes. Unipotent stem cells possess the most restricted potential, being capable of differentiating into a single type of mature cell, specific to a particular tissue or organ. An example of unipotent stem cells is the satellite cells in muscle tissue, which exclusively generate new muscle cells. Additionally, induced pluripotent stem cells (iPSCs) are genetically reprogrammed cells that share similar characteristics with pluripotent stem cells, capable of differentiating into various cell types in the body. Created with BioRender.com.

The adult human body consists of approximately 3.72 × 10^13^ cells that differ in shape and function [1]. Each cell specializes in performing a specific task depending on the type of tissue and the organ. Stem cells (SC) are cells with the ability to self-renew, differentiate into multiple cell types, and proliferate to produce and regenerate different types of tissues and organs. The main role of SCs is to maintain the tissue homeostasis of our body. Regarding their ability to differentiate, there are four types of stem cells: totipotent, pluripotent, multipotent, and unipotent stem cells (Figure 1). Lately, there has been an increased interest in SC since they can elucidate the development of the human body and help to better understand diseases. Novel research reveals an in vitro system approach for manipulating non-germline cells to induce totipotent stem cells and facilitate the development of multicellular organisms [2]. Understanding the multi-omics molecular background of stem cells could provide us with valuable information on disease treatment, including gynecological conditions and the production of healthy cells for use in regenerative medicine. Moreover, the outstanding results obtained from recent studies are truly promising. These results demonstrate a significant breakthrough in the field of stem cell research, as they showcase a person who has become HIV-free after undergoing a stem cell transplant. This groundbreaking achievement raises hopes for the potential use of stem cell therapies in the treatment of HIV and other diseases, opening up new possibilities for medical advancements and improved patient outcomes [3]. There have been lots of ongoing clinical trials and investigations with SCs so far, as well as published articles concerning different health conditions and disorders, such as traumatic brain injuries and various other neurological diseases, such as Alzheimer’s and Parkinson’s disease [4,5], stroke, traumatic brain injuries, learning defects, spinal cord injuries, missing teeth, amyotrophic lateral sclerosis, muscular dystrophy, myocardial infarction, diabetes, different cancers, Crohn’s disease, osteoarthritis, rheumatoid arthritis, wound healing, blindness, deafness, muscular dystrophies, etc. In this review article, we are going to emphasize the use of MSCs in the treatment of various demanding gynecological disorders, as well as discuss oogonial stem cell isolation and highlight the useful and promising role of mesenchymal stem cells (MSC) in this challenging issue [6,7].

## 2. Mesenchymal (Stromal) Stem Cells

Mesenchymal stem cells are adult SC and exist in almost all tissues. They have great potential for tissue regeneration. MSCs are a subtype of SCs and can be differentiated from other subtypes of SCs using several criteria. The first would be the expression of surface markers CD73 and CD105, as well as a lack of expression of markers CD14, CD34, and CD45. The other two criteria are the exhibition of plastic adherence in culture conditions and differentiation into osteoblasts, chondrocytes, and adipocytes [8,9].

**Figure 2:**
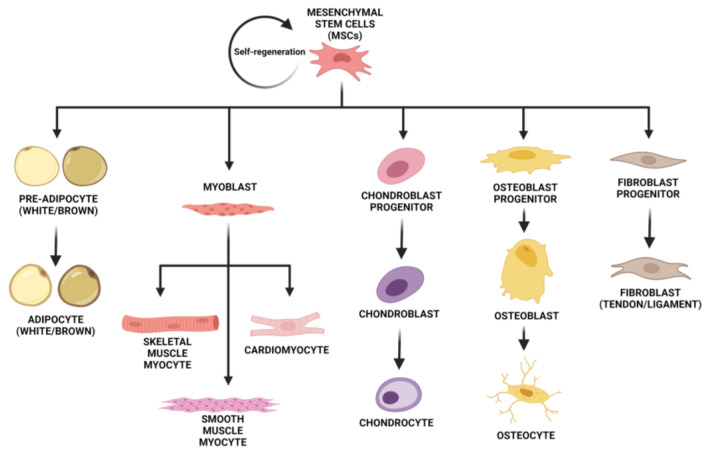
The differentiation capacity of MSCs. This capacity includes adipose, muscle, cartilage, bone, and connective tissue. Created with BioRender.com.

In addition, MSCs have shown paracrine properties by promoting immunoregulatory, anti-inflammatory, anti-apoptotic, and anti-oxidative effects, which grants their aspect as immuno-tolerant agents [10,11]. They also have analgesic and angiogenic effects. There are multiple types of MSCs, such as bone marrow MSCs (BM-MSC), adipose-derived MSCs (AD-MSC), menstrual blood-derived MSCs (Men-MSC), endometrial MSCs (eMSCs), umbilical cord MSCs (UC-MSC), amniotic fluid MSCs (AF-MSC), amnion-derived MSCs, and placental MSCs (P-MSC) [12,13]. BM-MSCs are a group of cells from which not only any cell from the lymphoid or myeloid line can be formed, but they also have the capacity to differentiate into osteoblasts, adipocytes, chondroblasts, endometrial, and granulosa cells [12,14]. AD-MSCs are an abundant source of SCs and have significant advantages over BM-MSCs. Minimally invasive methods are needed for their collection, they have 500 times more regenerative cells than the equivalent amount of bone marrow, and they have a similar differentiation capacity as the rest of MSCs [12,15] (Figure 2). It has been shown that AD-MSCs improve ovarian function in mice after being exposed to radiation by promoting the formation of new blood vessels and ovarian follicles [12,16]. Its use was also proven in premature ovarian insufficiency (POI) in humans [17]. Menstrual blood-derived SCs (Men-MSC) were discovered in 2007. They can be collected from menstrual blood, so it is non-invasive [13]. UC-MSCs are a great source of SCs that can be differentiated into almost every type of mesodermal and some non-mesodermal cell types and serve for tissue repair and immune response regulation. Their collection is painless and non-invasive. Scientific research revealed that UC-MSCs can increase ovarian function and lower ovarian cell death, and activate primordial follicles, which was proven in both animal and human models [12,18]. AF-MSCs are a mixed population of stem cells that have also shown promising data among different therapeutic and surgical applications. They express both adult and embryonic cell markers, indicating their stage is between embryonic and adult phenotype, which allows them to differentiate into cells of all three germ layers, including hepatic, myogenic, osteogenic, and neurogenic cell types [19,20,21]. P-MSCs can be isolated from chorionic villi early in gestation or at the time of birth. They have a higher immunomodulatory capacity in comparison to MSCs from other sources, as well as neuroprotective potential and benefits of autologous therapy for congenital diseases [22].

Stem cell research is one of the most proponent fields of research in medicine today. MSCs are actively being explored as potential therapeutic modalities across many fields of medicine, which has, in turn, led to many new discoveries in both scientific and clinical contexts. As an example, in a review by Liu J et al., the authors concluded that MSCs can slow down disease progression in some cases but accelerate it in others based on their interaction with the cellular microenvironment [23]. Another component of this review is the ongoing discovery of new stem cell populations, such as oogonial stem cells, with the goal of developing further treatment options.

### 2.1. Differential Phenotype Pattern of Mesenchymal Stem Cells in Male and Female Patients

Recent studies of mesenchymal stem cell heterogeneity based on immunophenotyping of stromal vascular fraction from lipoaspirates or micro fragmented lipoaspirates by polychromatic flow cytometry indicated a differential phenotype pattern of the applied MSC mixture in female and male patients [24]. Such information is extremely important in the treatment of gynecological diseases because stem cells of only female origin are mostly used in these studies. However, research will provide a better understanding of the heterogeneity of adipose MSC subpopulations used in the treatment of a variety of diseases, including many gynecological conditions.

### 2.2. Regenerative, Immunomodulatory, Anti-Inflammatory, and Antimicrobial Effect of Mesenchymal Stem Cells

MSCs are partially differentiated cells with the potential to specialize into more mature cells and, at the same time, self-replicate into daughter stem cells [25]. In addition to high proliferative potential and regenerative effect, MSCs are the source of several other effects on tissues, including growth support, self-renewal, immunomodulatory effect, and paracrine signaling effect. Recent research shows that, depending on the intercellular environment in which they are located, MSCs have an increased secretion of anti-inflammatory cytokines (IL-2, IL-4, IL-10, and TGF-β) and a decrease in pro-inflammatory cytokines (IL-1, IL-6, IL-17, and TNF-α) [26]. In a pro-inflammatory environment with high concentrations of TNF-α and IFN-γ, MSCs secrete anti-inflammatory mediators and become so-called MSC-2, which can inhibit the activation of dendritic cells, T and B lymphocytes, and NK cells [27].

MSCs can inhibit the activation and proliferation of NK cells and thus prevent cytotoxic immune response, while in the acquired immune defense, they inhibit the proliferation of B lymphocytes and the differentiation of T lymphocytes. β, bFGF, HGF, IL-6, SDF-1, M-CSF, VEGF, PIGF and MCP-1 [28]. MSCs also have antimicrobial or anti-inflammatory activity in general. MSCs secrete the antimicrobial factor lipocalin-2 and indoleamine 2,3-dioxygenase (IDO) in response to lipopolysaccharides, while unstimulated MSCs have been shown to inhibit the growth of gram-negative and gram-positive bacteria by secreting cathelicidin peptide LL-37 which interferes with bacterial cell membrane formation [29,30]. MSCs secrete β-defensin-2 and LL-37 from the umbilical cord and bone marrow, which confirms their antibacterial effects as well as the chemotactic, immunomodulatory, angiogenic, reparative, and anti-apoptotic effect of LL37 [31].

**Figure 3:**
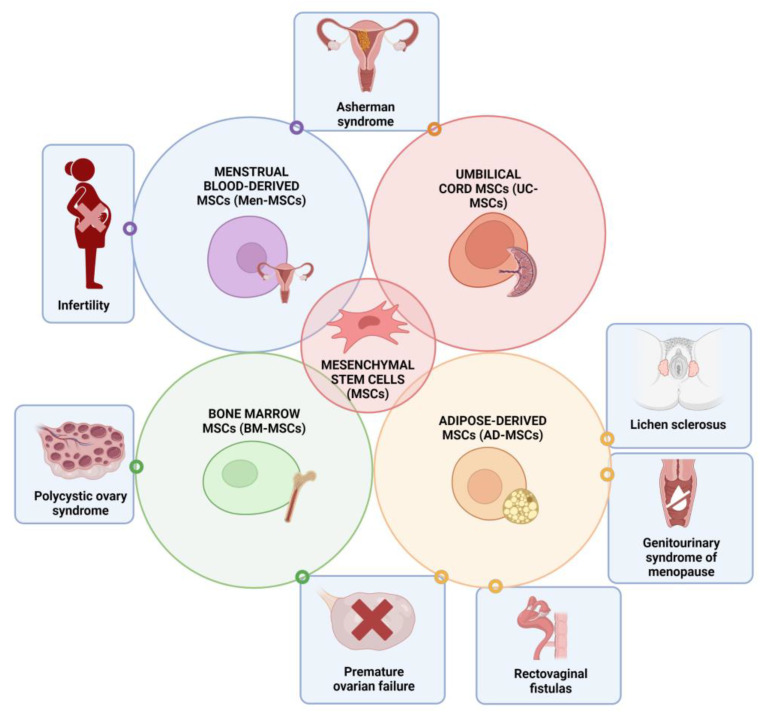
The therapeutic potential of MSCs in various conditions and diseases in gynecology. Created with BioRender.com.

The effects of MSCs have already been observed as treatment modalities in multiple problematic conditions. Such examples are the treatment of osteoarthritis and cartilage defects in orthopedics, as well as endodontics, periodontics, and oral surgery in stomatology [32,33,34,35,36]. MSCs have also found their application in tackling newer challenges, such as the COVID-19 pandemic [37]. In this review article, we will explore the regenerative, immunomodulatory, analgesic, and anti-inflammatory effects of mesenchymal stem cells in various conditions and diseases within gynecology, such as infertility, Asherman syndrome, lichen sclerosus, polycystic ovary syndrome, premature ovarian insufficiency, genitourinary syndrome of menopause, and rectovaginal fistulas. The aim is to assess their therapeutic potential in addressing these specific conditions and diseases in the future (Figure 3).

## 3. Application of Mesenchymal Stem Cells in Gynecological Disorders

### 3.1. Infertility

Infertility is one of the world’s most common gynecological disorders. It is the inability to conceive after six months of regular intercourse if the woman is older than 35 years of age or 12 months if she is younger than 35 years of age.

**Figure 4:**
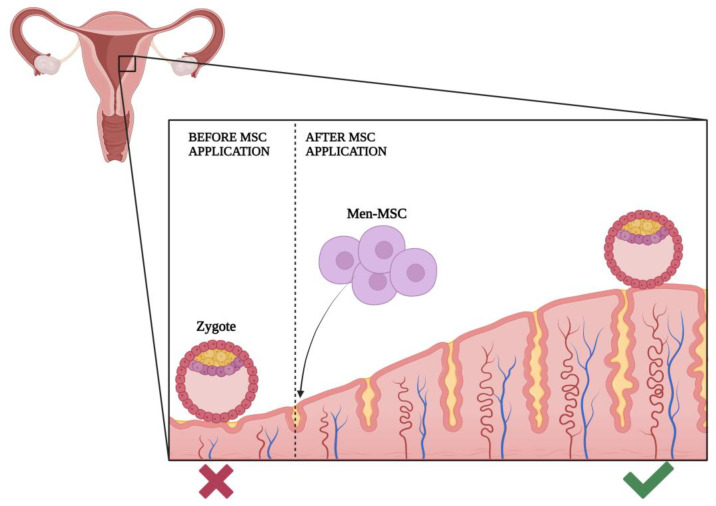
Endometrial restructuring achieved by Men-MSC therapy. Created with BioRender.com.

According to the literature, 15–20% of all couples will have some difficulties in achieving pregnancy during their reproductive life. The etiology of infertility is complex, and it can be caused by multiple diseases or conditions such as ovarian and ovulation disorders such as PCOS and POI, uterine origin including Asherman syndrome, tubal occlusions, cervical causes, endometriosis, premature menopause and older age, and anomalies of the reproductive system. Age is considered to be the most reliable predictor of fertility [38]. The most common causes of male infertility are low-quality semen, inability of sperm production, and obstructions of the male reproductive system. Every woman is born with a certain number of follicles and oocytes, the number of which gradually decreases during life due to atresia. Previous research proved that the incidence of infertility becomes notable at the age of 32 and rises to much higher values after the age of 37 [39]. Although age has been proven to affect the outcome of treatment using assisted reproductive technology (ART), different results are possible in women of the same reproductive age. It is known that obesity leads to a decrease in fertility. Fat tissue cells, adipocytes, release various adipokines and stimulate the body’s inflammatory reaction. This creates unfavorable conditions in the body, and previous research has shown a high frequency of anovulation, reduced oocyte quality, and endometrial receptivity in obese women [40]. This is why the results of ART are proven to be weaker, and the frequency of miscarriages and pregnancy-related complications such as preeclampsia and gestational diabetes is higher among obese women [40]. Ovarian reserve refers to the supply of ovarian primordial follicles. It is assessed by ultrasound and biochemical measurements. The antral follicle count (AFC) is measured by ultrasound from the second to the fifth day of the menstrual cycle, which includes the follicles of both ovaries with a size of two to ten millimeters (mm) in diameter [41]. Reference values for AFC vary, but it is approximately considered to be around three to nine per ovary. AFC usually depends on the age of the woman and decreases with age. Biochemical methods used to determine ovarian reserve are anti-Müllerian hormone (AMH) and follicle-stimulating hormone (FSH). Granulosa cells of pre-antral and antral follicles produce AMH. The concentration of AMH increases until the age of twenty-five and then decreases until menopause [41]. FSH is a gonadotropin that is synthesized and secreted by the adenohypophysis. It stimulates the growth and maturation of ovarian follicles, which creates a mature oocyte for ovulation. FSH is produced throughout life, but with exhausted ovarian folliculogenesis, when there are no more follicles that could respond to FSH, its level increases permanently. Elevated basal FSH values indicate a depleted ovarian reserve. Mesenchymal SCs were proven to renew ovarian reserves and improve endometrial function. Nevertheless, there is still an ongoing debate about the way it happens [12,42]. An important factor to take into account when discussing the advantages of MSCs in infertility treatment is their ability to be easily derived. In this context, Men-MSCs and AD-MSCs show an advantage, as their autologous transplantation less frequently results in graft rejection [42]. One of the MSCs that can be used to treat infertility is endometrial MSCs (eMSCs). The endometrium is part of the reproductive system that produces SCs particularly important for a woman’s reproductive life. The uterus, like most other organs in the body, has the ability to regenerate. With each menstrual cycle, the endometrial mucosa goes through a process of degeneration, cell proliferation, differentiation, and regeneration of the mucosa. Successful applications of adult stem cells to treat extensive intrauterine adhesions, endometrial atrophy (EA), and scarring might greatly improve menstruation and reprogram female fertility. Subendometrial transplantation of eMSCs can significantly increase the thickness of the endometrium and greatly improve in vitro fertilization outcomes [43]. Multipotent stem cells of the basal layer of the endometrium can self-renew, which enables the restoration of the functional layer of the endometrium. eMSCs cannot be easily and effectively collected and prepared, so alternatives had to be found. Another great source for treating infertility is Men-MSCs. Men-MSCs also have an endometrial origin, which is why they can be a good replacement. With the help of estrogen and progesterone, they rebuild endometrial tissue in vivo after differentiation into endometrial cells in vitro. It was also shown that infertility could be characterized by the expression of CD9, CD44, CD73, CD90, and CD166 markers. It has been shown that infertile women have higher expression of surface markers CD56 and SUSD2 on their Men-MSCs. Therefore, Men-MSCs can be used as ideal regenerative cells for treatment purposes such as endometrial repair in patients with intrauterine adhesions, improvement in ovarian functions in those with premature ovarian failure, and repair of patients with pelvic organ prolapse [43]. Men-MSCs are morphologically and functionally similar to cells derived directly from the endometrium; with dual expression of mesenchymal and embryonic cell markers, they proliferate and regenerate better than bone marrow mesenchymal stem cells. In addition, menstrual blood stem cells are extracted in a non-invasive and painless manner [44]. To study the properties of Men-MSCs, researchers analyzed the growth rate, cell surface marker expression, and proteome profile of Men-MSCs characteristics for healthy volunteers and patients with unexplained infertility. Additionally, the potential of Men-MSCs for decidualization and changes in gene expression, intracellular and secreted proteins during decidualization were revealed (Figure 4). Decidualization, or differentiation in the direction of the epithelium, is the restructuring of endometrial tissue necessary to create favorable conditions for the implantation of a fertilized oocyte. In order to use Men-MSCs for the treatment of infertility associated with endometrial disorders, it is necessary to elucidate the decidualization potential of these cells. Elevated progesterone and cellular cAMP levels after ovulation are known to activate the transcription factor Foxo1 in eMSCs, which halts the cell cycle, and cells differentiate into decidualized cells that control embryo implantation. It has also been shown that in vitro treatment of Men-MSCs with medium enriched with 8-bromo-cAMP and MPA successfully induced the decidualization process [44]. Human endometrial side population cells obtained by the side population technique are hormone-independent with intermediate telomerase activity and MSC phenotype, allowing for the neoformation of human endometrium in vivo [45]. In the same article, scientists explained that precursors of human decidual stromal cells from decidua-endometrial biopsies obtained from first-trimester pregnancies exhibited features compatible with perivascular endostromal stem cells (endoSSC, also expressing CD146 and PDGFR-b) in addition to the ability to decidualize in vitro, suggesting they are decidual MSCs involved in the mechanisms of maternal–fetal immune tolerance. Not only mesenchymal stromal cells are being used for clinical purposes but also their exosomes and mitochondria. MSC mitochondria are being used to improve oocyte quality [46].

### 3.2. Asherman Syndrome (AS)

Asherman syndrome is often a trauma-linked condition. It occurs when the basal layer of the endometrium is damaged either by physical trauma or after dilation and curettage. These situations trigger inflammation which predisposes the formation of adhesive bands from one side of the cavity to another. This is followed by an uncontrolled deposition of the extracellular matrix and fibrillary collagens. TGF is a central mediator of fibrogenesis and is found in greater amounts in damaged endometrium compared to normal endometrium [47]. Fibrotic factors such as TGF beta 1, alpha SMA, CTGF, and collagen I and III are crucial for the development of intrauterine adhesions, thus changing the uterine environment and reducing the regenerative capacity of endometrial mesenchymal stem cells [48]. Such a condition often causes infertility, habitual miscarriages, menstrual irregularities, chronic pain in the pelvis, and, during pregnancy, possible irregular positions of the placenta, such as placenta praevia or accreta. The main risk factor for the occurrence of AS are curettages, mostly performed after abortion, but also surgical procedures such as myomectomy, hysteroscopy, cesarean section, etc. Hysteroscopy is used as the gold standard in AS diagnostics. Hysterosalpingography and hystersonography can also serve as diagnostic options, but they are not as specific as hysteroscopy [47]. The prevalence of AS in infertile women is between 2 and 22% [49]. According to the European Society of Gynecological Endoscopy (ESGE), AS is classified into grades I–V according to hysteroscopy findings. The first line of therapy includes adhesiolysis performed by hysteroscopy, then progesterone and estrogen therapy for endometrial regeneration stimulation and barrier media to prevent re-adhesion. AS can be undiagnosed because routine diagnostic procedures such as ultrasound sometimes are not sensitive enough. A thin endometrium is present in 13% of women who had pregnancy loss in the first trimester and 30% of women undertaking curettage for a late abortion because of the destruction of the stratum basalis [50]. The executive pathway by which a therapeutic result is achieved is anti-inflammation and immunomodulation. MSCs, through their secretion of growth factors and cytokines, reduce tissue inflammation and promote regeneration. In research provided by Zhang et al. [51], seventeen women with Asherman syndrome were selected who met the following criteria: (a) women between 20 and 40 years of age, (b) women who had frozen embryos, (c) women who had two or more hysteroscopic adhesiolysis (HAS) with the return of uterine cavity to the normal state and (d) women whose endometrium did not expand to more than 5.5 mm while using 6–8 mg/day of estradiol with additional therapy. Those with congenital malformations and other damages to the endometrium, and other disorders that would compromise the implantation of the embryo, were excluded. The authors tried to regenerate endometrial tissue and enhance chances of pregnancy with transplantation of collagen scaffold/umbilical cord MSCs (CS/UC-MSC) to see if this therapy could help those where the conventional methods bore no results. After the transplantation, the thickness of the endometrium exceeded 5.5 mm in 12 patients, of which 6 patients’ endometrium exceeded 6 mm. Fifteen out of seventeen patients underwent 22 frozen-thawed embryo transfers (FET). Three became pregnant, two had babies without any complications and birth defects, and one aborted at 25+ weeks. Additionally, one of the women who did not undergo FET became pregnant naturally and had a healthy baby. The authors also checked endometrial microvascular density (MVD) factors and Ki67, ERα (estrogen receptor alpha), and PR (progesterone receptor), which were increased. All data showed that SC therapy can increase angiogenesis, proliferation, and differentiation in the endometrium [45]. In a meta-analysis provided by Zhao Y. et al. [52], which consisted of seven trials, 75 out of 77 patients, most of the patients treated with SC therapy noticed an improvement in their menstrual cycle within a few months. They also analyzed pregnancy outcomes for 66 women, and 65 of them became pregnant after the SC transplantation, while with conventional treatment, that was not possible. Regarding endometrial thickness, a total of 79 patients were included in analyses in eight studies. Change in thickness was noticed in 101 out of 105 patients, which is very promising for stem cell therapy [52]. In a study published by Jichun Tan I et al., the research observed the effect of MSCs obtained from menstrual blood on endometrial regeneration in women with AS. Seven infertile females with severe AS were part of this study. Patients were all of reproductive age (average 33.7 ± 1.5 years) with a mean infertile duration of 4.8 ± 1.2 years. The main cause of AS was a history of pregnancy-related curettages, including ectopic pregnancy surgery and artificial abortion. On day two of menses, sterile menstrual blood was collected to culture Men-MSCs. Autologous Men-MSCs were transplanted into the uterus on day 16. Transplanting the Men-MSCs into the wombs led to significant proliferation of the endometrium under estrogen stimulation in all patients. In five women, the thickness of the endometrium reached 7 mm, which was ready for embryo transfer. One patient had a spontaneous pregnancy. Embryo transfer was conducted in the remaining four patients, and two of them became pregnant. A total of three patients (43%) conceived successfully [49].

In a paper by Hailan Ma et al. [53], the authors evaluated the effects of intrauterine transplantation of Men-MSCs on endometrial thickness and pregnancy outcomes in patients with refractory Asherman syndrome. This study included a group of 12 infertile women 20 to 40 years of age who were resistant to conventional treatments. Autologous Men-MSCs were successfully isolated and expanded from menstrual blood and transplanted into the uterus of each patient, followed by hormonal stimulation. After 14 days of the transplantation transvaginal ultrasound, there was an improvement in the endometrial thickness: 3.9 ± 0.9 to 7.5 ± 0.6 mm (*p* < 0.001). Five patients achieved pregnancy, four after embryo transfers, and one from natural conception.

### 3.3. Polycystic Ovary Syndrome (PCOS)

The main characteristics of PCOS are oligoovulation or anovulation, clinical or biochemical hyperandrogenism, and polycystic ovarian morphology detected by ultrasound. According to the Rotterdam criteria, the diagnosis of PCOS is established if two out of three criteria are met, with confirmation that differential diagnostic conditions such as congenital adrenal hyperplasia, hypothalamic-pituitary disorders, androgen-secreting tumors, and Cushing syndrome are excluded [54]. Women suffering from PCOS have a higher risk of developing numerous diseases such as cardiovascular disease, type 2 diabetes, obesity and glucose intolerance, insulin resistance, and metabolic syndrome [55]. The exact etiology of the disease is still unknown, but research shows that low-grade chronic inflammation and oxidative stress are involved in the pathogenesis of PCOS [56]. The first line of treatment for this disease is combined oral hormonal contraception. Other therapeutic options include metformin, inositol, antiandrogens, and others. It is today’s most common reproductive disorder, affecting 4–18% of women of reproductive age [57,58]. R. Man Chugh et al. [57] suggested that using MSC therapy could improve metabolic disorders and rehabilitate ovaries for pregnancy. In their in vitro research, they used human BM-MSCs on androgen-producing H295R cells and studied the expression of the androgen-producing gene. In the vivo model, they used human BM-MSCs on a letrozole-induced mouse model of PCOS. As a result, they saw a remarkably suppressed expression of the steroidogenic gene and restored fertility in test animals. It was revealed that it was cytokine IL-10 (interleukine-10), an anti-inflammatory cytokine, that was responsible for this good response and that it could reverse this disorder [59]. They performed other research in which they tried to prove another regulatory mechanism of BM-MSCs. It was found that BM-MSCs secrete bone morphogenetic proteins (BMP)–molecules that can regulate the production of androgen. H295R cells were used again and showed notable suppression of androgen production, androgen-synthesizing genes, and inflammatory gene expression. When BM-MSCs were applied with a BMP-2 gene knockdown on H295R cells, it showed higher expression of genes for the production of androgen, which shows BMP-2 is part of the secretome responsible for therapeutic characteristics of BM-MSCs in PCOS [57]. Those findings still have to be confirmed by clinical trials in humans. Z. Kalhori et al. studied the improvement in folliculogenesis by transplantation of BM-MSCs in mice with induced polycystic ovary syndrome. Every day for five weeks, mice were injected with testosterone enanthate (1 mg/100 g/body weight subcutaneous), BM-MSCs were labeled with Hoechst 33,342 (0.5 mg/mL) and then injected into the mice at 1 and 14 days after PCOS confirmation. They discovered a significant increase in the total volume of the ovary, cortex, number of antral follicles, volume of oocytes, and zona pellucida thickness. Furthermore, there was a significant decrease in the number of primary and preantral follicles. A significant increase was seen in the serum levels of FSH and total antioxidant capacity and a significant decrease in the serum levels of testosterone, LH, tumor necrosis factor-alpha (TNF-alfa), and IL-6 [60].

Inflammatory cytokines present in granulosa cells are thought to lead to poor oocyte quality. Therefore, the anti-inflammatory effect of mesenchymal stem cells leads to the development of oocytes of better quality and, ultimately, to a better pregnancy outcome for PCOS patients. This effect is mediated by growth factors and suppressive cytokines, which regulate and eventually reduce the inflammatory process. In research provided by Zhao et al., they reported the anti-inflammatory effect presented in UC-MSC-derived exosomes specifically on the granulosa cells in the follicular fluid of the PCOS patients, and it was discovered that TNF-α and interferon-gamma (IFN-γ) were significantly decreased and that IL-10 levels were increased in the PCOS patients [61].

**Figure 5:**
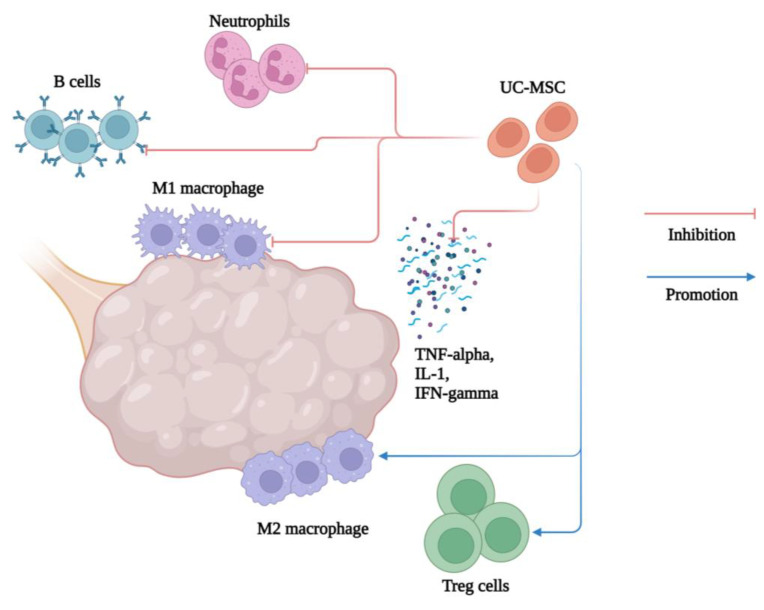
Anti-inflammatory and immunomodulatory effects of UC-MSCs in the treatment of PCOS. Created with BioRender.com.

Xie Q. et al. investigated how the administration of human (h) UC-MSCs reduces inflammation in mice with PCOS induced by dehydroepiandrosterone (DHEA). Moreover, they found that the implication of MSCs significantly downregulated the expression of pro-inflammatory factors (TNF-α, IL-1β, and IFN-γ) and fibrosis-related genes (CTGF) in ovarian and uterus tissues and affected the systemic inflammatory response. The percentage of peripheral neutrophils, M1 macrophages, and B cells was significantly reduced, while M2 macrophages and regulatory T cells (Tregs) were increased in hUC-MSC-treated mice. In the spleen, the percentage of neutrophils, M1 macrophages, IFN-γ + CD19 + B cells, IFN-γ + CD4 + T cells (Th1), and IL-17 + CD4 + T cells (Th17) was significantly decreased in hUC-MSC-treated mice (Figure 5) [62].

### 3.4. Primary Ovarian Insufficiency (POI)

Primary ovarian insufficiency or premature ovarian failure [63] is a disorder of the menstrual cycle and ovaries that represents itself as premature menopause due to decreased ovarian function, although it is different from menopause due to some women still having occasional periods and rarely even getting pregnant. POI appears in women younger than 40 and, in total, affects about 1% of the world women population. The criteria required for diagnosis are menstrual cycle disorders such as amenorrhea or oligomenorrhea and elevated FSH values in two consecutive measurements with an interval of 4 weeks [64]. Very often, it is a devastating condition for relatively young women who did not fulfill their reproductive ability, and until recently, ovarian regeneration was not possible. Patients with POI may present with secondary amenorrhea or oligomenorrhea and symptoms of estrogen deficiency such as night sweats and hot flashes in waves. In addition, estrogen deficiency causes vaginal atrophy, and patients may suffer from dyspareunia. Furthermore, sleep disorders, mood changes, brain fog, reduced libido, and loss of concentration are often present. The two leading mechanisms of POI are follicular depletion and follicular dysfunction. The primordial follicle consists of the primary oocyte surrounded by follicular cells. At birth, there are around one to two million primordial follicles, and by puberty, their number decreases to 300,000. Follicular depletion implies a condition in which no primordial follicles are present in the ovary, while in follicular dysfunction, the number of follicles in the ovaries is normal, but their function is disturbed. The etiology is not yet clear. Common causes are chromosomal and genetic abnormalities such as fragile X or Turner syndrome, family history, autoimmune diseases, metabolic disorders, infections, and environmental factors such as different toxins, smoking, or diet. If POI is caused by gynecological surgery, chemotherapy, or radiotherapy, it is classified as secondary or iatrogenic POI. However, in 90% of cases, the cause is still unknown [65]. Early diagnosis and initiation of treatment are necessary because POI leads to an increased incidence of cardiovascular diseases and a decrease in bone mineral density. Today, hormone replacement therapy is used as the first-line treatment for patients with POI.

In the last decade, there have been many ongoing types of research on using MSCs as a treatment for this disorder with promising results. After the transplantation, MSCs migrate to the ovaries through a process called “homing”, and their cytokines stop the inflammation and promote the proliferation of damaged tissue. This process represents one of the main executive pathways of MSCs as therapeutic agents, including both anti-inflammation and immunomodulation. The ability of MSCs to control and downregulate the inflammation directly affects the etiology of this condition. Most of the used MSCs, such as BM-MSCs and UC-MSCs, settled in the medulla or hilum of the ovaries, while a smaller number settled in the cortex. It was shown that, by their secretome, they promote the formation and growth of follicles and possibly have the capacity to restore the menstrual cycle. Once they are settled, they can be located alive in the ovaries even after a couple of months which is a positive sign and gives great hope for further exploration [62,66]. Research conducted by Ling L. et al. showed hAD-MSC (human) transplantation reduced ovarian injury and improved ovarian function in rats with POI. Compared to the POI group, the AMH level was significantly higher in the hAD-MSC-treated group, starting from the second week after hAD-MSC transplantation, while the FSH level was significantly lower. In the fourth week after hAD-MSC transplantation, the E2 level was significantly higher in the hAD-MSC treated group than it was in the POI group. Furthermore, compared to the POI group, the number of secondary, primary, and primordial follicles was significantly greater in the hAD-MSC-treated group, while the number of atretic follicles was significantly lower. The results showed that hAD-MSCs secreted G-CSF, FGF2, IGF-1, HGF, and VEGF and especially expressed high levels of FGF2, IGF-1, HGF, and VEGF. These growth factors secreted by hAD-MSCs inhibit chemotherapy-induced granulosa cell apoptosis, promote angiogenesis, and regulate follicular development. This study demonstrates hAD-MSC transplantation improves ovarian function in rats with POI at least partly through a paracrine mechanism [67].

Yin et al. noted an increased production of cytokines such as transforming growth factor beta (TGF-β), which suppress inflammation and contribute to the recovery of ovarian function. They also observed a concomitant decrease in interferon-gamma (IFN-γ), which inhibits ovulation and promotes follicular atresia. Two weeks after hP-MSC (human placental) therapy, the number of estrous cycles was increased, as well as estrogen production, along with a decrease in FSH, thus confirming recovery of ovarian endocrine function. Additionally, they described an increase in blood flow and a decrease in apoptosis of granulosa cells in the mice after hP-MSC transplantation [68]. Mohamed et al. demonstrated that administration of UC-MSC into the ovaries after chemotherapy-induced POI mice results in improved ovarian function and an increase in fertility. After therapy, there was a significant decrease in FSH levels and an increase in serum AMH levels [69].

A study presented by Sun M. et al. revealed AD-MSCs resumed ovarian function after chemotherapy-induced ovarian injury. AD-MSCs could increase follicle and oocyte counts. AD-MSCs treatment also decreased granulosa cell apoptosis [70]. In a study of 30 women with POI, Gabr et al. [71] administered granulocyte colony-stimulating factor (G-CSF) followed by isolation of autologous BM-MSCs, which were injected into the ovarian artery and ovarian tissue under laparoscopic guidance. A total of 86.7% of women with POI showed a decrease in FSH levels and a rise in estrogen and AMH levels after 4 weeks of injection. Ovulation was observed in 18 patients (60%) with ovum sizes ranging from 12–20 mm. One patient had spontaneous pregnancy, while three patients were subjected to IVF cycles. In addition, Wang et al. showed that Men-MSCs produced a high level of fibroblast growth factor 2, which enhanced cell survival, proliferation, and function to repair tissue damage. They concluded that Men-MSCs repair ovarian injury, improve ovarian function, and stimulate regeneration [72].

### 3.5. Genitourinary Syndrome of Menopause (GSM)

Genitourinary atrophy, previously known as vulvovaginal atrophy (VVA) or urogenital atrophy, nowadays called genitourinary syndrome of menopause (GSM), is a syndrome characterized by a lack of estrogen and therefore vaginal dryness, dyspareunia, often vulvovaginitis (VV), cystitis, urinary incontinence, burning, itching, stranguria, lesser sensitivity, etc. The vaginal microbiota is very important for women’s gynecologic and reproductive health and acts by preventing colonization by pathogenic organisms, including sexually transmitted and urinary tract infectious agents [73]. Vaginal health is associated with an environment rich in Lactobacillus species, while a lack of Lactobacillus is associated with gynecologic infections, vaginal dryness, and VV. Women with GSM are more often found to have a bacterial flora that is relatively lower in Lactobacillus. Estrogen not only improves vaginal symptoms but also allows re-colonization of the postmenopausal vagina with Lactobacillus [73]. Estrogen also contributes to the deposition of glycogen in the vaginal epithelium, which is metabolized by indigenous bacterial communities to produce the organic acids needed to protect the genital tract. VVA is highly prevalent, affecting up to 84% of menopausal women, depending on the study [74]. Two studies have been carried out that show the prevalence of GSM. In a cross-sectional, population-based study of US women aged 40 to 65 years, symptoms consistent with VVA occurred in 57% of sexually active women [74]. In a study of 913 menopausal women presenting for routine examinations, GSM was diagnosed in 65% of women one year before menopause and in 85% of women six years after menopause [74,75]. These symptoms are very uncomfortable, and although they are not life-threatening or dangerous, they greatly impact the quality of life. Since the lifespan of humans, and especially women, is extended, most women will live a third, and many of them even half of their lives in postmenopause. That is a very serious fact because quality of life is very important, especially in such a long life. So far, there are numerous treatments available for GSM, but none of them are completely satisfying and long-lasting. For these reasons, recent studies have explored the treatment of vaginal atrophy using more specific, non-hormonal alternative therapies [76]. Apart from these novel alternative agents, MSCs are known to have anti-inflammatory and regenerative effects [76]. G. Casarotti and C. Tremolada [77] published interesting results considering a new treatment based on a single local vaginal application of autologous micro-fragmented adipose tissue (MFAT). They patented a special technology called Lipogems^®^ which processes adipose tissue and removes it together with blood and oil residues but leaves AD-MSCs. Lipogems^®^ is a device that efficiently harvests, processes, and transfers adipose tissue with unique characteristics, including long-term expression and secretion of physiologically active concentrations of angiogenic, immunomodulatory, anti-inflammatory, anti-apoptotic, and anti-fibrotic secretome [78]. The resulting Lipogems product is composed of small, specifically sized ‘adipose’ clusters of adipocytes maintaining an intact perivascular environment and a pericytes’ activation through ball-bearing-induced mechanical shock. Lipogems^®^ creates a minimally manipulated fat-derived product according to the regulations set forth by US Food and Drug Administration (FDA) [78]. It has received FDA clearance as a class II medical device for the processing of autologous adipose tissue. According to the FDA, we could define Lipogems^®^ as, first, autologous; second, minimally manipulated; third, intended for homologous use; fourth, enzyme-free; fifth, not dependent on the metabolic activity of the cells for its primary function; sixth, used in the same surgical procedure, and, seventh, not combined with anything other than saline [78]. AD-MSCs are considered to have a key role in tissue recovery. This is, in part, considered to be a result of their significant differentiation potential into adipocytes, osteoblasts, chondrocytes, and others. Secondly, AD-MSCs have the ability to secrete growth factors and different cytokines and chemokines, which induce and assist the regenerative process in tissues [79]. Casarotti et al. performed a case series with 35 patients, 42–66 years old, for three consecutive years. The main inclusion criteria for patients were (a) menopause for at least four years, (b) never have taken hormone replacement therapy (HRT) or local estrogens, (c) have severe GSM symptoms, and (d) have at least four of the following symptoms—vaginal dryness, dyspareunia, recurrent cystitis, stinging-bladder tenesmus, and any level of urinary incontinence. Before the application of AD-MSCs, 57% of patients described their symptoms as severe and 43% as moderate. Six months after the treatment, 54% had mild symptoms, and the rest of them (46%) were without any symptoms, which was an astonishing result. Over the next two years, which is a very long and satisfying period, only three patients described mild symptoms, and the rest stayed asymptomatic [77]. These results in GSM treatment, compared to all others so far available, such as laser therapy, radiofrequency, magnetic stimulation, local estrogen therapy, and others, establish MSC as the longest-lasting therapy, and since there were no side-effects described, completely safe as well.

Lipogems^®^ was also demonstrated to be effective in the relief of symptoms of postmenopausal genitourinary atrophy [77] in three patients who remained symptom-free and showed both clinical and histopathological complete recovery for three years. A biopsy of the urogenital area demonstrated a completely normal-looking epithelial mucosa consistent with a premenopausal condition [79].

### 3.6. Lichen Sclerosus (LS)

Lichen sclerosus is a chronic disease with possible autoimmune etiology. Clinically, it presents as a chronic inflammatory skin disease. LS is associated with a potentially greater degree of scarring and a higher risk of cancer in the anogenital region if it is not treated [80]. LS can appear in all ages and genders. It is usually diagnosed in postmenopausal women, although the disease already occurs in about 50% of affected women prior to menopause [81]. There is also genetic predisposition, as approximately 10% of patients with LS have relatives with the same disease. In the case of vulvar LS, an autoimmune phenotype has been observed, which involves dense T-cell infiltration, increased levels of Th1-specific cytokines, and enhanced BIC/miR-155 expression as well as auto-antibodies against extracellular matrix protein 1 and BP180 antigen [81]. Women with LS have an increased prevalence of HLA-DQ 7, -DQ 8, -DQ 9, and -DR 12 compared to controls, with 50% of adult females and 66% of prepubertal females expressing HLA-DQ7 [81]. On the contrary, HLA-DR17 shows a negative association with LS, so these HLA antigens and their associated haplotypes could play a role in susceptibility and protection from LS [82]. The exact etiology of LS remains unknown, although a strong connection between autoimmune disease and familial occurrence has been recorded. LS as an inflammatory disease is supported by gene expression profiles and is mediated by the upregulation of T-helper type 1 cytokine. There is a fortified association between Th1 responses and autoimmune diseases. MicroRNA-155 (miR-155) is known to be involved in the promotion of Th1 differentiation. When microRNA is overexpressed, it can disrupt suppression mediated by T regulatory cells (Treg) and trigger a loss of self-tolerance and promote inflammation and eventually induce autoimmunity. Increased collagen synthesis is also associated with dysregulation because of overexpression and can lead to sclerotic tissue formation. In addition, miR-155 inhibits tumor suppressor genes FOXO3 and CDKN1B, leading to even more collagen synthesis [81]. Clinical presentation of LS can vary from asymptomatic to highly symptomatic. The connection between the aspect and/or type of lesion with the severity of the disease is difficult to prove. Women with the symptomatic disease most often complain of burning and pruritus or soreness, and these symptoms usually worsen at night [83]. Vulvar irritation is the most frequent symptom reported in adults [84]. Due to intense and repetitive scratching, because of pruritus, a lichen simplex sclerosus may develop. In addition to the above symptoms, other symptoms such as dyspareunia, dysuria, pain and soreness, voiding dysfunction, and bleeding may also be present [83]. As the disease progresses, scratching and sclerotic changes lead to pain from erosions and fissures [83]. Eventually, progressive scarring and tissue adhesion result in the narrowing of the introitus, which is caused by loss of elasticity and then easily tears at the base of the posterior fourchette [84]. Plaques of ivory white, atrophic, or thickened skin with ecchymoses and hemorrhage from repeated scratching of the sclerotic and thinned labia minora is the characteristic clinical appearance of LS [83]. The crinkled texture change is classic and, generally, pathognomonic [84]. Around 75% of all vulvar cancers are associated with vulvar inflammatory conditions, such as LS [82]. Although LS is not premalignant, it has been systematically associated with an increased risk of keratinized vulvar squamous carcinoma, estimated at 2.6–6.7% [83]. It is characterized by hypopigmentation, skin atrophy, sclerotic plaques, and extreme itch, most commonly in the genital area. Eshtiaghi et al. [85] performed a review of several studies, including 15 women who were treated with AD-MSCs and Platelet-Rich Plasma (PRP), a cohort of 36 patients treated with only AD-MSCs, and a trial with 8 women who were also treated with AD-MSCs. In all groups, a significant amelioration of symptoms was noticed—their life quality and sexual function were significantly approved after this treatment [85]. AD-MSCs are supposed to be able to restore and regenerate damaged tissue and are now purported to have tissue regenerative potential in fibrotic conditions [85]. AD-MSCs within lipoaspirates are reported to be able to proliferate and differentiate into various mesenchymal tissues, and they also have anti-inflammatory and immunomodulatory properties [85]. The great therapeutic potential is explained by the presence of a high concentration of pluripotent mesenchymal stem cells, endothelial progenitor cells, T-cells, B-cells, mast cells, and adipose-resident macrophages and a vast array of bioactive secretory factors that are collectively referred to as adipose-derived stromal vascular fraction (AD-SVF) [84]. MSCs residing in AD-SVF demonstrated an anti-inflammatory effect on immune cell responses by interacting with dendritic cells, T-lymphocytes, and natural killer cells [86]. MSC may also perform immunosuppressive functions by secreting immunomodulatory factors, which switch off T-cell surveillance and chronic inflammatory processes [86]. Platelet-rich plasma (PRP) promotes the healing process of tissue by stimulating the release of cytokines and growth factors [82]. The effectiveness of PRP is based upon the high level of growth factors such as PDGF, TGF-B, and EGF, which modulate mesenchymal cell proliferation and extracellular matrix synthesis [82]. Tedesco et al. [86] published a study on 40 patients (M = 24; F = 16, with a mean age of 43 years (18 to 78 years), randomized into two arms using a 1:1 allocation ratio and a computer-generated random numbers table. Inclusion criteria were age over 18, histopathologic diagnosis of lichen sclerosus, good general conditions, and proven failure of previous treatment. All patients (AD-SFV and AD-SFV plus PRP) experienced a reduction in symptoms and an improvement in elasticity, hydration, and atrophy of the affected skin and mucosae [86]. A total of 13 patients reported progressive symptoms decreased until they disappeared; 23 patients showed significant improvement in symptoms; four patients manifested no changes, and interestingly, two patients also referred to a reduction of white lesions (hypochromic areas) [86]. Overall, separated evaluation of the AD-SVF and AD-SVF plus patients demonstrated no statistical difference in the mean clinical score (AD-SVF = 2.0 ± 0.8; AD-SVF plus PRP = 1.8 ± 1.1, *p* = 0.42) [86].

### 3.7. Rectovaginal Fistulas

Rectovaginal fistulas are abnormal connections between the rectum and vagina. They can be congenital or acquired—very often during or after vaginal childbirth, abscesses, various infections, malignancies, and inflammatory bowel diseases such as Chron’s disease and ulcerative colitis [87]. Treatment of rectovaginal fistulas is challenging and very often unsuccessful. The treatment of patients in which complete closure cannot be achieved despite the combination of biological therapy and surgery is still not well defined [88]. These patients may benefit from innovative therapeutic approaches such as mesenchymal stromal cells (MSCs) [87]. Bone marrow and adipose tissue are the most readily available sources of MSCs, and adipose tissue is preferable because of its abundance, easy access, and simple isolation procedure [88]. The use of AD-MSCs, either culture-expanded or obtained by mechanical or enzymatic treatment as a stromal vascular fraction, created a large interest as both in vitro and in vivo studies confirmed their anti-inflammatory and regenerative properties [88]. Micro fragmented adipose tissue injection has been recently tested in a series of 19 patients with complex idiopathic anal fistulas as first-line therapy or as a “rescue” therapy after the failure of surgical repair [88]. In research completed by Cao Y. et al. [89], 1252 patients were divided into two groups (placebo and stem cell group), and it was shown that the stem cell group had more than 20% higher healing percentage than the placebo group [89]. In another analysis completed by García-Arranz M et al. [90], they tried to determine the use of allogeneic AD-MSCs in treating rectovaginal fistulas in Chron’s disease in 10 patients. A total of 20 million SCs were injected into the submucosa of the vaginal walls, and the healing was evaluated after 12 weeks. If there was little effect from the first dose, another 40 million SCs were injected into the site. After 52 weeks, during a follow-up, sixty percent (60%) of patients had a complete recovery—vaginal and rectal sides were re-epithelialized, and there was no vaginal drainage. This procedure was concluded to be safe with a promising success rate [90].

MFAT was demonstrated to be effective after a single injection in a case series of 15 patients with perianal fistulas with Crohn’s disease [78]. Patients were treated with a single local injection of MFAT [78]. After six weeks, 10 patients showed complete clinical and instrumental remission, four patients slightly improved, and only one patient showed no improvement [78]. De Weerd et al. [91] performed a pilot study on six patients with recalcitrant RVF, four due to obstetric injury and two associated with Crohn’s disease. The fat graft from the lower abdomen was injected transperineally around the fistula tract [91]. At the end of the injection procedure, the fistula tract was transected transversely [91]. In one patient, the fistula healed after a single treatment, while the other five required two treatments with a 6-week interval [91]. In the patients with an RVF due to obstetric injury, no recurrence occurred during follow-up, mean of 41 months (range 4–53) [90]. In the two patients with Crohn’s disease, a new fistula developed after 23 and 25 months, respectively [91]. The main finding of this pilot study is the successful use of autologous fat grafting in the treatment of recalcitrant RVF. Based on their results, the authors concluded that autologous fat grafting can become a new and promising technique in the treatment of recalcitrant RVF, and viable tissue between the vagina and rectum can be obtained with the use of this technique [91]. Unlike other procedures, this technique does not include a wide dissection or an increased risk of neurovascular injury, which could lead to dyspareunia and incontinence, and is therefore reduced. Fat harvesting is associated with minimal morbidity.

**Figure 6:**
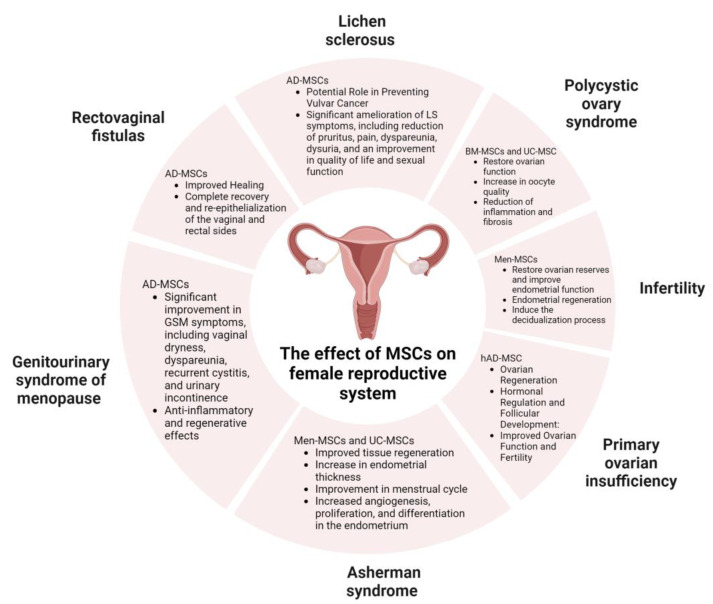
A graphical overview of the therapeutic effects of MSCs in various gynecological conditions. Created with BioRender.com.

The potential of SCs in medicine is overwhelmingly positive (Figure 6). Through mechanisms of regeneration, differentiation, immunomodulation, angiogenesis, anti-oxidation, and anti-apoptosis, they have led to new treatment modalities and successful treatment of many difficult diseases. In gynecology, in particular, examples of these conditions are infertility, PCOS, Asherman syndrome, GSM, rectovaginal fistulas, lichen sclerosus, and premature ovarian failure. Additionally, the use of MFAT containing MSCs in treating rectovaginal fistula (RVF) related to Chron’s disease in combination with surgical procedure is a promising approach in such a delicate, complicated, and until now, very difficult medical condition to solve with often very poor results [92]. The aforementioned conditions are complicated to manage and severely negatively impact the patient’s quality of life, a fact that emphasizes the clinical importance of this research, as well as its impact on modern medicine.

## 4. Oogonial Stem Cells

Oogonial stem cells (OSCs) are a special subclass of stem cells with the ability to regenerate oocytes and replenish the primary follicle reserve. In recent years, studies working with OSCs have challenged the rule that defines a finite number of oocytes from birth. [93]. However, the existence of OSCs is currently still under debate.

OSCs have been isolated in multiple mammalian species thus far. MacDonald JA et al. described OSC characteristics from mice ovaries [94]. The study marked several consistencies between mice and humans in this regard, mainly the existence of OSC subpopulations that differ in genetic expression. The authors also noted that the subpopulations of OSCs isolated at 3–20 months of age are present throughout adult life. However, over time these cells lose expression of DPPA3, a gene responsible for differentiation into oocytes, which could explain the loss of oogenesis following age. Likewise, in a study published by Wang C et al., OSCs were isolated from a neonatal piglet [95]. The authors managed to obtain evidence in support of OSC existence by stimulating differentiation into oocyte-like cells using porcine follicular fluid and retinoic acid.

On the other hand, some studies argue against the existence of OSCs in our species. In a study published by Wagner M et al., a single-cell analysis was completed to observe the different cell subpopulations in the human ovarian cortex [96]. The authors reported the profiles of over 24,000, with the assumption that OSCs will be isolated using the DDX4 antibody. However, DDX4 isolation only yielded perivascular cells, while no traces of OSCs were found. As a result, the study does not support the existence of these cells and reinforces the idea of a limited reserve of oocytes. The isolation method using the DDX4 antibody marker is traditionally used for OSCs but has recently sparked debate over the identity of the isolated cells, whether they are truly OSCs or simply oocytes [97]. A new method published by Sequeira RC et al. uses a combination of DDX4 and IFITM3 antibodies coupled with magnetic-activated cell sorting. The study produced a significantly higher count of OSCs and yielded results that support the findings of OSC research.

On balance, this field is one that warrants further research. The successful isolation of OSCs could lead to a revolution in the field of gynecology and the potential treatment of conditions discussed in this review, such as infertility. Additionally, in the context of MSCs, a study by Mirzaeian et al. relates the process of oogenesis from MSCs to the specific interaction between MSCs and OSCs [98].

## 5. Epithelial–Mesenchymal Transition in the Context of Mesenchymal Stem Cells

When analyzing the topic of stem cells in gynecology, one of the points worth discussing is the process known as epithelial–mesenchymal transition (EMT). What this process entails is epithelial cells acquiring certain properties of mesenchymal cells. This process is an integral component of embryonal growth, wound healing, and tissue regeneration. However, it has also been observed in pathological processes such as pathological fibrosis and cancer progression [99]. In this context, EMT is considered to be a result of interactions between cancer cells and MSCs in the tumor microenvironment [99]. A study by So KA et al. [100] observed the interaction between MSCs and cancer cells on a molecular level. The results of the study established a critical role of interleukin-6 in oncogenic EMT. On the other hand, a paper by Lin X et al. [101] links the induction of EMT in gynecological cancer to the dysregulation of long non-coding RNA. A third paper, by Du J et al., establishes hypoxia as a potential EMT inducer in ovarian cancer [102].

The oncological component of EMT is of great interest in the field of gynecology. For example, tumor heterogeneity in ovarian cancer can, in part, be found due to tumor cells going through EMT. There is also evidence that genetic variants can lead to EMT in endometrial cancer [103]. A meta-analysis by Muhammad JA et al. [104] investigated EMT as a potentially poor prognostic factor in gynecological cancers. More specifically, the authors observed the correlation between tumor budding (TB), which is defined as the finding of single or cluster tumor cells at the invasive front and is considered a poor prognostic factor, and EMT. The authors concluded that there is a high correlation between these two factors, which links EMT to poor prognosis.

Understanding the pathogenic role and mechanism of induction of EMT gives rise to potential treatment modalities based on these processes. There are several EMT markers in gynecological cancers, such as E-cadherin, N-cadherin, alpha-catenin, beta-catenin, and vimentin. The expression of E-cadherin and its transcriptional repressor TWIST1 has been demonstrated as markers indicating an epithelial–mesenchymal transition (EMT) in metastatic tumors originating from different primary sites [105]. These markers are discussed as potential therapeutic targets in these cancers [106]. However, treatment in this area has not yet been perfected. Cancer stem cells (CSC), a subpopulation of cancer cells, control the tumor microenvironment in order to promote tumor growth, angiogenesis, invasion, metastasis, and resistance to treatment. One of the executive mechanisms of these cells is considered to be EMT [107]. It is necessary to discuss all aspects in which SCs can influence various pathologies, including negative ones. Understanding the processes of EMT is important for comprehending the differentiation potential of stem cells, as well as understanding the possible malignant potential that stem cells possess. Recent scientific research indicates the safety of using mesenchymal stem cells, while the use of totipotent stem cells potentially carries the risk of malignant transformation [108]. Understanding the EMT process is particularly crucial for the development of potential new molecular targets for therapeutic interventions that would inhibit cellular signals associated with EMT [109].

## 6. Conclusions

In conclusion, recent studies revealed the great medical potential of stem cells in the treatment of various gynecological diseases. The results of a wide range of research, both in animal models and clinical trials in humans, showed that the use of mesenchymal stem cells is safe, has long-term and promising results, and provides multiple regenerative, angiogenic, immunomodulatory, analgesic, and antimicrobial (anti-inflammatory) effects. The use of stem cells, with emphasis on MSCs, in gynecology and medicine, in general, is very promising due to their high efficiency, safety, and the possibility of wide application in numerous cases and complicated disorders which are challenging and to this date have not been successfully and long-lastingly treated. Future investigations are needed to determine the exact way of their impact on the human body, standardization of type and quantity needed, and facilitate accessibility and application for all.

## Data Availability

No new data were created or analyzed in this study. Data sharing is not applicable to this article.

## References

[1] Bianconi E., Piovesan A., Facchin F., Beraudi A., Casadei R., Frabetti F., Vitale L., Pelleri M.C., Tassani S., Piva F. (2013). An estimation of the number of cells in the human body. Ann. Hum. Biol..

[2] Hu Y., Yang Y., Tan P., Zhang Y., Han M., Yu J., Zhang X., Jia Z., Wang D., Yao K. (2023). Induction of mouse totipotent stem cells by a defined chemical cocktail. Nature.

[3] Reardon S. (2023). Third patient free of HIV after receiving virus-resistant cells. Nature.

[4] Danielyan L., Beer-Hammer S., Stolzing A., Schafer R., Siegel G., Fabian C., Kahle P., Biedermann T., Lourhmati A., Buadze M. (2014). Intranasal delivery of bone marrow-derived mesenchymal stem cells, macrophages, and microglia to the brain in mouse models of Alzheimer’s and Parkinson’s disease. Cell Transplant..

[5] Zhao L., Liu J.W., Shi H.Y., Ma Y.M. (2021). Neural stem cell therapy for brain disease. World J. Stem Cells.

[6] Dulak J., Szade K., Szade A., Nowak W., Jozkowicz A. (2015). Adult stem cells: Hopes and hypes of regenerative medicine. Acta Biochim. Pol..

[7] Yamanaka S. (2020). Pluripotent Stem Cell-Based Cell Therapy-Promise and Challenges. Cell Stem Cell.

[8] Lv F.J., Tuan R.S., Cheung K.M., Leung V.Y. (2014). Concise review: The surface markers and identity of human mesenchymal stem cells. Stem Cells.

[9] Ullah I., Subbarao R.B., Rho G.J. (2015). Human mesenchymal stem cells—current trends and future prospective. Biosci. Rep..

[10] Brown C., McKee C., Bakshi S., Walker K., Hakman E., Halassy S., Svinarich D., Dodds R., Govind C.K., Chaudhry G.R. (2019). Mesenchymal stem cells: Cell therapy and regeneration potential. J. Tissue Eng. Regen. Med..

[11] Han Y., Li X., Zhang Y., Han Y., Chang F., Ding J. (2019). Mesenchymal Stem Cells for Regenerative Medicine. Cells.

[12] Esfandyari S., Chugh R.M., Park H.S., Hobeika E., Ulin M., Al-Hendy A. (2020). Mesenchymal Stem Cells as a Bio Organ for Treatment of Female Infertility. Cells.

[13] Zhao Y.X., Chen S.R., Su P.P., Huang F.H., Shi Y.C., Shi Q.Y., Lin S. (2019). Using Mesenchymal Stem Cells to Treat Female Infertility: An Update on Female Reproductive Diseases. Stem Cells Int..

[14] Gao L., Huang Z., Lin H., Tian Y., Li P., Lin S. (2019). Bone Marrow Mesenchymal Stem Cells (BMSCs) Restore Functional Endometrium in the Rat Model for Severe Asherman Syndrome. Reprod. Sci..

[15] Lee R.H., Kim B., Choi I., Kim H., Choi H.S., Suh K., Bae Y.C., Jung J.S. (2004). Characterization and expression analysis of mesenchymal stem cells from human bone marrow and adipose tissue. Cell Physiol. Biochem..

[16] Terraciano P., Garcez T., Ayres L., Durli I., Baggio M., Kuhl C.P., Laurino C., Passos E., Paz A.H., Cirne-Lima E. (2014). Cell therapy for chemically induced ovarian failure in mice. Stem Cells Int..

[17] Sen Halicioglu B., Saadat K., Tuglu M.I. (2022). Adipose-Derived Mesenchymal Stem Cell Transplantation in Chemotherapy-Induced Premature Ovarian Insufficiency: The Role of Connexin and Pannexin. Reprod. Sci..

[18] Mohamed S.A., Shalaby S., Brakta S., Elam L., Elsharoud A., Al-Hendy A. (2019). Umbilical Cord Blood Mesenchymal Stem Cells as an Infertility Treatment for Chemotherapy Induced Premature Ovarian Insufficiency. Biomedicines.

[19] Baughn C., Campion S., Elbabaa S. (2022). Amniotic fluid-derived stem cell potential for therapeutic and surgical use: A review of the literature. Prenat. Diagn..

[20] Borlongan C.V. (2017). Amniotic fluid as a source of engraftable stem cells. Brain Circ..

[21] Miki T. (2011). Amnion-derived stem cells: In quest of clinical applications. Stem Cell Res. Ther..

[22] Kabagambe S., Keller B., Becker J., Goodman L., Pivetti C., Lankford L., Chung K., Lee C., Chen Y.J., Kumar P. (2017). Placental mesenchymal stromal cells seeded on clinical grade extracellular matrix improve ambulation in ovine myelomeningocele. J. Pediatr. Surg..

[23] Liu J., Gao J., Liang Z., Gao C., Niu Q., Wu F., Zhang L. (2022). Mesenchymal stem cells and their microenvironment. Stem Cell Res. Ther..

[24] Zenic L., Polancec D., Hudetz D., Jelec Z., Rod E., Vidovic D., Staresinic M., Sabalic S., Vrdoljak T., Petrovic T. (2021). Polychromatic Flow Cytometric Analysis of Stromal Vascular Fraction from Lipoaspirate and Microfragmented Counterparts Reveals Sex-Related Immunophenotype Differences. Genes.

[25] Zakrzewski W., Dobrzynski M., Szymonowicz M., Rybak Z. (2019). Stem cells: Past, present, and future. Stem Cell Res. Ther..

[26] Dabrowska S., Andrzejewska A., Janowski M., Lukomska B. (2020). Immunomodulatory and Regenerative Effects of Mesenchymal Stem Cells and Extracellular Vesicles: Therapeutic Outlook for Inflammatory and Degenerative Diseases. Front. Immunol..

[27] Lopez-Garcia L., Castro-Manrreza M.E. (2021). TNF-alpha and IFN-gamma Participate in Improving the Immunoregulatory Capacity of Mesenchymal Stem/Stromal Cells: Importance of Cell-Cell Contact and Extracellular Vesicles. Int. J. Mol. Sci..

[28] Fan X.L., Zhang Y., Li X., Fu Q.L. (2020). Mechanisms underlying the protective effects of mesenchymal stem cell-based therapy. Cell Mol. Life Sci..

[29] Sharma A., Chakraborty A., Jaganathan B.G. (2021). Review of the potential of mesenchymal stem cells for the treatment of infectious diseases. World J. Stem Cells..

[30] Zhang Q.Y., Yan Z.B., Meng Y.M., Hong X.Y., Shao G., Ma J.J., Cheng X.R., Liu J., Kang J., Fu C.Y. (2021). Antimicrobial peptides: Mechanism of action, activity and clinical potential. Mil. Med. Res..

[31] Pahar B., Madonna S., Das A., Albanesi C., Girolomoni G. (2020). Immunomodulatory Role of the Antimicrobial LL-37 Peptide in Autoimmune Diseases and Viral Infections. Vaccines.

[32] Smojver I., Katalinić I., Bjelica R., Gabrić D., Matišić V., Molnar V., Primorac D. (2022). Mesenchymal Stem Cells Based Treatment in Dental Medicine: A Narrative Review. Int. J. Mol. Sci..

[33] Molnar V., Pavelić E., Vrdoljak K., Čemerin M., Klarić E., Matišić V., Bjelica R., Brlek P., Kovačić I., Tremolada C. (2022). Mesenchymal Stem Cell Mechanisms of Action and Clinical Effects in Osteoarthritis: A Narrative Review. Genes.

[34] Molnar V., Pavelić E., Jeleč Ž., Brlek P., Matišić V., Borić I., Hudetz D., Rod E., Vidović D., Starčević N. (2023). Results of Treating Mild to Moderate Knee Osteoarthritis with Autologous Conditioned Adipose Tissue. J. Pers. Med..

[35] Polancec D., Zenic L., Hudetz D., Boric I., Jelec Z., Rod E., Vrdoljak T., Skelin A., Plecko M., Turkalj M. (2019). Immunophenotyping of a Stromal Vascular Fraction from Microfragmented Lipoaspirate Used in Osteoarthritis Cartilage Treatment and Its Lipoaspirate Counterpart. Genes.

[36] Primorac D., Hudetz D., Borić I., Jeleč Ž., Rod E., Vrdoljak T., Lauc G., Skelin APolančec D., Zenić L., Trbojević-Akmačić I. (2018). Cell therapy in treatment of cartilage tissue defects: Experiences from St. Catherine Specialty Hospital. Paediatr. Croat..

[37] Primorac D., Stojanović Stipić S., Strbad M., Girandon L., Barlič A., Frankić M., Ivić I., Marasović Krstulović D., Jukić I., Halassy B. (2021). Compassionate mesenchymal stem cell treatment in a severe COVID-19 patient: A case report. Croat. Med. J..

[38] Crawford N.M., Steiner A.Z. (2015). Age-related infertility. Obstet. Gynecol. Clin. N. Am..

[39] Practice Committee of the American Society for Reproductive Medicine (2013). Definitions of infertility and recurrent pregnancy loss: A committee opinion. Fertil. Steril..

[40] Silvestris E., de Pergola G., Rosania R., Loverro G. (2018). Obesity as disruptor of the female fertility. Reprod. Biol. Endocrinol..

[41] Bedenk J., Vrtacnik-Bokal E., Virant-Klun I. (2020). The role of anti-Mullerian hormone (AMH) in ovarian disease and infertility. J. Assist. Reprod. Genet..

[42] Rungsiwiwut R., Virutamasen P., Pruksananonda K. (2021). Mesenchymal stem cells for restoring endometrial function: An infertility perspective. Reprod. Med. Biol..

[43] Lv Q., Wang L., Luo X., Chen X. (2021). Adult stem cells in endometrial regeneration: Molecular insights and clinical applications. Mol. Reprod. Dev..

[44] de Miguel-Gomez L., Lopez-Martinez S., Frances-Herrero E., Rodriguez-Eguren A., Pellicer A., Cervello I. (2021). Stem Cells and the Endometrium: From the Discovery of Adult Stem Cells to Pre-Clinical Models. Cells.

[45] Ugurlu B., Karaoz E. (2020). Comparison of similar cells: Mesenchymal stromal cells and fibroblasts. Acta Histochem..

[46] Skliute G., Bausyte R., Borutinskaite V., Valiuliene G., Kaupinis A., Valius M., Ramasauskaite D., Navakauskiene R. (2021). Menstrual Blood-Derived Endometrial Stem Cells’ Impact for the Treatment Perspective of Female Infertility. Int. J. Mol. Sci..

[47] Benor A., Gay S., DeCherney A. (2020). An update on stem cell therapy for Asherman syndrome. J. Assist. Reprod. Genet..

[48] Hu J., Zeng B., Jiang X., Hu L., Meng Y., Zhu Y., Mao M. (2015). The expression of marker for endometrial stem cell and fibrosis was increased in intrauterine adhesious. Int. J. Clin. Exp. Pathol..

[49] Tan J., Li P., Wang Q., Li Y., Li X., Zhao D., Xu X., Kong L. (2016). Autologous menstrual blood-derived stromal cells transplantation for severe Asherman’s syndrome. Hum. Reprod..

[50] Smikle C., Yarrarapu S.N.S., Khetarpal S. (2021). Asherman Sindrome StatPearls.

[51] Zhang Y., Shi L., Lin X., Zhou F., Xin L., Xu W., Yu H., Li J., Pan M., Pan Y. (2021). Unresponsive thin endometrium caused by Asherman syndrome treated with umbilical cord mesenchymal stem cells on collagen scaffolds: A pilot study. Stem Cell Res. Ther..

[52] Zhao Y., Luo Q., Zhang X., Qin Y., Hao J., Kong D., Wang H., Li G., Gu X., Wang H. (2020). Clinical Efficacy and Safety of Stem Cell-Based Therapy in Treating Asherman Syndrome: A System Review and Meta-Analysis. Stem Cells Int..

[53] Ma H., Liu M., Li Y., Wang W., Yang K., Lu L., He M., Deng T., Li M., Wu D. (2020). Intrauterine transplantation of autologous menstrual blood stem cells increases endometrial thickness and pregnancy potential in patients with refractory intrauterine adhesion. J. Obstet. Gynaecol. Res..

[54] Wang R., Mol B.W. (2017). The Rotterdam criteria for polycystic ovary syndrome: Evidence-based criteria?. Hum. Reprod..

[55] Diamanti-Kandarakis E., Dunaif A. (2012). Insulin resistance and the polycystic ovary syndrome revisited: An update on mechanisms and implications. Endocr. Rev..

[56] Murri M., Luque-Ramirez M., Insenser M., Ojeda-Ojeda M., Escobar-Morreale H.F. (2013). Circulating markers of oxidative stress and polycystic ovary syndrome (PCOS): A systematic review and meta-analysis. Hum. Reprod. Update.

[57] Chugh R.M., Park H.S., Esfandyari S., Elsharoud A., Ulin M., Al-Hendy A. (2021). Mesenchymal Stem Cell-Conditioned Media Regulate Steroidogenesis and Inhibit Androgen Secretion in a PCOS Cell Model via BMP-2. Int. J. Mol. Sci..

[58] Dumesic D.A., Abbott D.H., Sanchita S., Chazenbalk G.D. (2020). Endocrine-Metabolic Dysfunction in Polycystic Ovary Syndrome: An Evolutionary Perspective. Curr. Opin. Endocr. Metab. Res..

[59] Chugh R.M., Park H.S., El Andaloussi A., Elsharoud A., Esfandyari S., Ulin M., Bakir L., Aboalsoud A., Ali M., Ashour D. (2021). Mesenchymal stem cell therapy ameliorates metabolic dysfunction and restores fertility in a PCOS mouse model through interleukin-10. Stem Cell Res. Ther..

[60] Kalhori Z., Azadbakht M., Soleimani Mehranjani M., Shariatzadeh M.A. (2018). Improvement of the folliculogenesis by transplantation of bone marrow mesenchymal stromal cells in mice with induced polycystic ovary syndrome. Cytotherapy.

[61] Prayitno G.D., Lestari K., Sartika C.R., Djuwantono T., Widjaya A., Muharam R., Hidayat Y.M., Wulandari D., Haifa R., Naura N.F. (2022). Potential of Mesenchymal Stem Cells and Their Secretomes in Decreasing Inflammation Markers in Polycystic Ovary Syndrome Treatment: A Systematic Review. Medicines.

[62] Xie Q., Xiong X., Xiao N., He K., Chen M., Peng J., Su X., Mei H., Dai Y., Wei D. (2019). Mesenchymal Stem Cells Alleviate DHEA-Induced Polycystic Ovary Syndrome (PCOS) by Inhibiting Inflammation in Mice. Stem Cells Int..

[63] Welt C.K. (2008). Primary ovarian insufficiency: A more accurate term for premature ovarian failure. Clin. Endocrinol..

[64] Webber L., Davies M., Anderson R., Bartlett J., Braat D., Cartwright B., Cifkova R., de Muinck Keizer-Scharma S., European Society for Human R, Embryology Guideline Group on POI (2016). ESHRE Guideline: Management of women with premature ovarian insufficiency. Hum. Reprod..

[65] Maclaran K., Panay N. (2015). Current concepts in premature ovarian insufficiency. Womens Health.

[66] Li Z., Zhang M., Tian Y., Li Q., Huang X. (2021). Mesenchymal Stem Cells in Premature Ovarian Insufficiency: Mechanisms and Prospects. Front. Cell Dev. Biol..

[67] Ling L., Feng X., Wei T., Wang Y., Wang Y., Wang Z., Tang D., Luo Y., Xiong Z. (2019). Human amnion-derived mesenchymal stem cell (hAD-MSC) transplantation improves ovarian function in rats with premature ovarian insufficiency (POI) at least partly through a paracrine mechanism. Stem Cell Res. Ther..

[68] Yin N., Zhao W., Luo Q., Yuan W., Luan X., Zhang H. (2018). Restoring Ovarian Function With Human Placenta-Derived Mesenchymal Stem Cells in Autoimmune-Induced Premature Ovarian Failure Mice Mediated by Treg Cells and Associated Cytokines. Reprod. Sci..

[69] Mohamed S.A., Shalaby S.M., Abdelaziz M., Brakta S., Hill W.D., Ismail N., Al-Hendy A. (2018). Human Mesenchymal Stem Cells Partially Reverse Infertility in Chemotherapy-Induced Ovarian Failure. Reprod. Sci..

[70] Sun M., Wang S., Li Y., Yu L., Gu F., Wang C., Yao Y. (2013). Adipose-derived stem cells improved mouse ovary function after chemotherapy-induced ovary failure. Stem Cell Res. Ther..

[71] Gabr H., Elkheir W.A., El-Gazzar A. (2016). Autologus stem cell transplantation in patients with idiopathic premature ovarian failure. J. Tissue Sci. Eng..

[72] Wang Z., Wang Y., Yang T., Li J., Yang X. (2017). Study of the reparative effects of menstrual-derived stem cells on premature ovarian failure in mice. Stem Cell Res. Ther..

[73] Kagan R., Kellogg-Spadt S., Parish S.J. (2019). Practical Treatment Considerations in the Management of Genitourinary Syndrome of Menopause. Drugs Aging.

[74] Shifren J.L. (2018). Genitourinary Syndrome of Menopause. Clin. Obstet. Gynecol..

[75] Angelou K., Grigoriadis T., Diakosavvas M., Zacharakis D., Athanasiou S. (2020). The Genitourinary Syndrome of Menopause: An Overview of the Recent Data. Cureus.

[76] Kasap B., Kasap S., Vatansever S., Kendirci R., Yilmaz O., Calisir M., Edgunlu T., Akin M.N. (2019). Effects of adipose and bone marrow-derived mesenchymal stem cells on vaginal atrophy in a rat menopause model. Gene.

[77] Casarotti G., Tremolada C. (2020). A new treatment of genito-urinary post-menopausal atrophy with autologous micro-fragmented fat tissue: A thirty-six months follow up case series. Eur. Rev. Med. Pharmacol. Sci..

[78] Tremolada C., Allegri M., Slevin M. (2019). Mesenchymal Stromal Cells and Micro Fragmented Adipose Tissue: New Horizons of Effectiveness of Lipogems. J. Stem Cell Res. Dev..

[79] Xu T., Yu X., Yang Q., Liu X., Fang J., Dai X. (2019). Autologous Micro-Fragmented Adipose Tissue as Stem Cell-Based Natural Scaffold for Cartilage Defect Repair. Cell Transplant..

[80] Chamli A., Souissi A. (2021). Lichen Sclerosus.

[81] Kirtschig G. (2016). Lichen Sclerosus-Presentation, Diagnosis and Management. Dtsch. Arztebl. Int..

[82] Krapf J.M., Mitchell L., Holton M.A., Goldstein A.T. (2020). Vulvar Lichen Sclerosus: Current Perspectives. Int. J. Womens Health.

[83] Perez-Lopez F.R., Vieira-Baptista P. (2017). Lichen sclerosus in women: A review. Climacteric.

[84] Murphy R. (2010). Lichen sclerosus. Dermatol. Clin..

[85] Eshtiaghi P., Sadownik L.A. (2019). Fact or Fiction? Adipose-Derived Stem Cells and Platelet-Rich Plasma for the Treatment of Vulvar Lichen Sclerosus. J. Low. Genit. Tract. Dis..

[86] Tedesco M., Bellei B., Garelli V., Caputo S., Latini A., Giuliani M., Cota C., Chichierchia G., Romani C., Foddai M.L. (2020). Adipose tissue stromal vascular fraction and adipose tissue stromal vascular fraction plus platelet-rich plasma grafting: New regenerative perspectives in genital lichen sclerosus. Dermatol. Ther..

[87] Debeche-Adams T.H., Bohl J.L. (2010). Rectovaginal fistulas. Clin. Colon. Rectal Surg..

[88] Laureti S., Gionchetti P., Cappelli A., Vittori L., Contedini F., Rizzello F., Golfieri R., Campieri M., Poggioli G. (2020). Refractory Complex Crohn’s Perianal Fistulas: A Role for Autologous Microfragmented Adipose Tissue Injection. Inflamm. Bowel Dis..

[89] Cao Y., Su Q., Zhang B., Shen F., Li S. (2021). Efficacy of stem cells therapy for Crohn’s fistula: A meta-analysis and systematic review. Stem Cell Res. Ther..

[90] Garcia-Arranz M., Herreros M.D., Gonzalez-Gomez C., de la Quintana P., Guadalajara H., Georgiev-Hristov T., Trebol J., Garcia-Olmo D. (2016). Treatment of Crohn’s-Related Rectovaginal Fistula With Allogeneic Expanded-Adipose Derived Stem Cells: A Phase I-IIa Clinical Trial. Stem Cells Transl. Med..

[91] de Weerd L., Weum S., Norderval S. (2015). Novel treatment for recalcitrant rectovaginal fistulas: Fat injection. Int. Urogynecol. J..

[92] Dimova A., Erceg Ivkošić I., Brlek P., Dimov S., Pavlović T., Bokun T., Primorac D. Case report: Novel approach in rectovaginal fistula treatment- the combination of modified Martius flap and microfragmented adipose tissue (Submitted).

[93] Meng L., Zhang Y., Hua Y., Ma Y., Wang H., Li X., Jiang Y., Zhu G. (2023). Identification of oogonial stem cells in chicken ovary. Cell Prolif..

[94] MacDonald J.A., Sheehan H.C., Piasecki A., Faustino L.R., Hauschildt C., Stolzenbach V., Woods D.C., Tilly J.L. (2023). Characterization of Oogonial Stem Cells in Adult Mouse Ovaries with Age and Comparison to In Silico Data on Human Ovarian Aging. Stem Cells Dev..

[95] Wang C., Sun Q., Li S., Liu G., Ren J., Li Y., Ding X., Zhu J., Dai Y. (2023). Isolation of female germline stem cells from neonatal piglet ovarian tissue and differentiation into oocyte-like cells. Theriogenology.

[96] Wagner M., Yoshihara M., Douagi I., Damdimopoulos A., Panula S., Petropoulos S., Lu H., Pettersson K., Palm K., Katayama S. (2020). Single-cell analysis of human ovarian cortex identifies distinct cell populations but no oogonial stem cells. Nat. Commun..

[97] Sequeira R.C., Sittadjody S., Criswell T., Atala A., Jackson J.D., Yoo J.J. (2021). Enhanced method to select human oogonial stem cells for fertility research. Cell Tissue Res..

[98] Mirzaeian L., Eivazkhani F., Saber M., Moini A., Esfandiari F., Valojerdi M.R., Fathi R. (2023). In-vivo oogenesis of oogonial and mesenchymal stem cells seeded in transplanted ovarian extracellular matrix. J. Ovarian Res..

[99] Chen T., You Y., Jiang H., Wang Z.Z. (2017). Epithelial-mesenchymal transition (EMT): A biological process in the development, stem cell differentiation, and tumorigenesis. J. Cell Physiol..

[100] So K.A., Min K.J., Hong J.H., Lee J.K. (2015). Interleukin-6 expression by interactions between gynecologic cancer cells and human mesenchymal stem cells promotes epithelial-mesenchymal transition. Int. J. Oncol..

[101] Lin X., Qiu J., Hua K. (2018). Long non-coding RNAs as emerging regulators of epithelial to mesenchymal transition in gynecologic cancers. Biosci. Trends.

[102] Du J., Sun B., Zhao X., Gu Q., Dong X., Mo J., Sun T., Wang J., Sun R., Liu Y. (2014). Hypoxia promotes vasculogenic mimicry formation by inducing epithelial-mesenchymal transition in ovarian carcinoma. Gynecol. Oncol..

[103] Bilyk O., Coatham M., Jewer M., Postovit L.M. (2017). Epithelial-to-Mesenchymal Transition in the Female Reproductive Tract: From Normal Functioning to Disease Pathology. Front. Oncol..

[104] Ailia M.J., Thakur N., Chong Y., Yim K. (2022). Tumor Budding in Gynecologic Cancer as a Marker for Poor Survival: A Systematic Review and Meta-Analysis of the Perspectives of Epithelial-Mesenchymal Transition. Cancers.

[105] Brlek P., Bukovac A., Kafka A., Pecina-Slaus N. (2021). TWIST1 upregulation affects E-cadherin expression in brain metastases. Clin. Transl. Oncol..

[106] Zhou X.M., Zhang H., Han X. (2014). Role of epithelial to mesenchymal transition proteins in gynecological cancers: Pathological and therapeutic perspectives. Tumour Biol..

[107] Keyvani V., Riahi E., Yousefi M., Esmaeili S.A., Shafabakhsh R., Moradi Hasan-Abad A., Mahjoubin-Tehran M., Hamblin M.R., Mollazadeh S., Mirzaei H. (2022). Gynecologic Cancer, Cancer Stem Cells, and Possible Targeted Therapies. Front. Pharmacol..

[108] Wang Y., Yi H., Song Y. (2021). The safety of MSC therapy over the past 15 years: A meta-analysis. Stem Cell Res. Ther..

[109] Bukovac A., Kafka A., Raguz M., Brlek P., Dragicevic K., Muller D., Pecina-Slaus N. (2021). Are We Benign? What Can Wnt Signaling Pathway and Epithelial to Mesenchymal Transition Tell Us about Intracranial Meningioma Progression. Cancers.

